# The contribution of NADPH thioredoxin reductase C (NTRC) and sulfiredoxin to 2-Cys peroxiredoxin overoxidation in *Arabidopsis thaliana* chloroplasts

**DOI:** 10.1093/jxb/eru512

**Published:** 2015-01-05

**Authors:** Leonor Puerto-Galán, Juan M. Pérez-Ruiz, Manuel Guinea, Francisco Javier Cejudo

**Affiliations:** Instituto de Bioquímica Vegetal y Fotosíntesis, Universidad de Sevilla-CSIC, Avda Américo Vespucio, 49, 41092-Sevilla, Spain

**Keywords:** *Arabidopsis thaliana*, chloroplast, 2-Cys peroxiredoxin, overoxidation, redox regulation, sulfiredoxin, thioredoxin reductase.

## Abstract

Hydrogen peroxide is a harmful by-product of photosynthesis, which also has important signalling activity. Therefore, the level of hydrogen peroxide needs to be tightly controlled. Chloroplasts harbour different antioxidant systems including enzymes such as the 2-Cys peroxiredoxins (2-Cys Prxs). Under oxidizing conditions, 2-Cys Prxs are susceptible to inactivation by overoxidation of their peroxidatic cysteine, which is enzymatically reverted by sulfiredoxin (Srx). In chloroplasts, the redox status of 2-Cys Prxs is highly dependent on NADPH-thioredoxin reductase C (NTRC) and Srx; however, the relationship of these activities in determining the level of 2-Cys Prx overoxidation is unknown. Here we have addressed this question by a combination of genetic and biochemical approaches. An *Arabidopsis thaliana* double knockout mutant lacking NTRC and Srx shows a phenotype similar to the *ntrc* mutant, while the *srx* mutant resembles wild-type plants. The deficiency of NTRC causes reduced overoxidation of 2-Cys Prxs, whereas the deficiency of Srx has the opposite effect. Moreover, *in vitro* analyses show that the disulfide bond linking the resolving and peroxidatic cysteines protects the latter from overoxidation, thus explaining the dominant role of NTRC on the level of 2-Cys Prx overoxidation *in vivo*. The overoxidation of chloroplast 2-Cys Prxs shows no circadian oscillation, in agreement with the fact that neither the *NTRC* nor the *SRX* genes show circadian regulation of expression. Additionally, the low level of 2-Cys Prx overoxidation in the *ntrc* mutant is light dependent, suggesting that the redox status of 2-Cys Prxs in chloroplasts depends on light rather than the circadian clock.

## Highlight

This work shows the dominant effect of NADPH thioredoxin reductase C (NTRC) over sulfiredoxin on 2-Cys peroxiredoxin (2-Cys Prx) overoxidation, and uncovers an NTRC-independent, light-dependent component contributing to 2-Cys Prx overoxidation in *Arabidopsis thaliana* chloroplasts.

## Introduction

Aerobic metabolism inevitably produces reactive oxygen species (ROS) including singlet oxygen, superoxide anions, or hydrogen peroxide. These species are highly oxidizing and may have a toxic effect, though they also exert important signalling activity. Hydrogen peroxide affects the expression of a large set of genes in plants (Vandenabeele *et al.*, 2003; Vanderauwera *et al.*, 2005), and is involved in different signal transduction pathways (Pitzschke *et al.*, 2006; Laloi *et al.*, 2007). In order to balance the toxic and signalling activities of hydrogen peroxide, its intracellular concentration needs to be tightly regulated. In this regard the activity of 2-Cys peroxiredoxins (2-Cys Prxs) has been proposed to exert a relevant function. 2-Cys Prxs are thiol-based peroxidases classified as typical and atypical, depending on whether they have dimeric or monomeric structures, respectively (Rhee and Woo, 2011). Both typical and atypical 2-Cys Prxs have a common reaction mechanism, which depends on two cysteine residues, termed peroxidatic and resolving. The thiolate form of the peroxidatic cysteine reacts with the peroxide and becomes transiently oxidized as sulfenic acid (-SOH), which then reacts with the resolving cysteine to render both cysteines oxidized as a disulfide bridge (Rhee *et al.*, 2012). For a new catalytic cycle, this disulfide has to be reduced in a reaction catalysed by a protein disulfide reductase, which is most frequently thioredoxin (Trx), though glutaredoxins (Grx) and cyclophilins might also be involved (Dietz, 2011). However, in the presence of hydrogen peroxide, the sulfenic intermediate may become overoxidized to sulfinic (-SO_2_H) or even sulfonic (-SO_3_H) acid leading to the inactivation of the enzyme (Yang *et al.*, 2002). 2-Cys Prx overoxidation not only causes the inactivation of the peroxidase activity, but also induces the oligomerization and chaperone activity of the enzyme (Jang *et al.*, 2004). The sulfinic form of 2-Cys Prxs can be converted back into the sulfenic form in an ATP-dependent reaction catalysed by sulfiredoxin (Srx) (Biteau *et al.*, 2003; Woo *et al.*, 2003).

In eukaryotic organisms, the interaction of the peroxidatic and resolving cysteines is limited by two motifs, GGLG and YF, at the C-terminal region of the 2-Cys Prxs; this structural constraint makes the sulfenic intermediate more susceptible to overoxidation (Wood *et al.*, 2003). It has been proposed that the inactivation of 2-Cys Prxs by overoxidation results in a gain of function of the eukaryotic enzymes promoting a further increase of hydrogen peroxide concentration, which may thus be used for signalling purposes: the so-called floodgate hypothesis (Wood *et al.*, 2003). In contrast, 2-Cys Prxs of prokaryotic organisms, with the exception of some cyanobacterial strains (Pascual *et al.*, 2010), are less sensitive to inactivation by overoxidation so that hydrogen peroxide is efficiently detoxified and has less relevance as a second messenger in these organisms. More recently, the circadian oscillation of 2-Cys Prx overoxidation has been shown, which persists without transcription in mammalian and algal cells (O’Neill and Reddy, 2011; O’Neill *et al.*, 2011). The extension of these analyses to other organisms led to the proposal that 2-Cys Prx overoxidation constitutes a conserved marker of circadian rhythms, thus linking the redox status of the cell with the circadian clock (Edgar *et al.*, 2012).

In plants, Prxs are encoded by a gene family formed by 9–10 genes (Dietz, 2003). Remarkably, four of these Prxs, typical 2-Cys Prx A and 2-Cys Prx B, and atypical Prx Q and Prx IIE, are localized in the chloroplast in *Arabidopsis thaliana* (Dietz, 2003), typical 2-Cys Prxs being among the most abundant proteins of this organelle (Dietz *et al.*, 2006). As in other organisms, the catalytic cycle of chloroplast 2-Cys Prxs requires the reduction of the disulfide bridge linking the peroxidatic and resolving cysteines. The Trx-like CDSP32 (Broin *et al.*, 2002), Trx *x* (Collin *et al.*, 2003) and an NADPH thioredoxin reductase (NTR) with a joint Trx domain at the C-terminus, termed NTRC (Serrato *et al.*, 2004), have been proposed as putative reductants of 2-Cys Prxs in chloroplasts. NTRC, which is exclusive to oxygenic photosynthetic organisms, is localized in plastids (Serrato *et al.*, 2004; Moon *et al.*, 2006; Kirchsteiger *et al.*, 2012) and assays *in vitro* revealed that it is a very efficient reductant of 2-Cys Prxs (Moon *et al.*, 2006; Pérez-Ruiz *et al.*, 2006; Alkalfioui *et al*., 2007; Pérez-Ruiz and Cejudo, 2009); this has been further supported by analyses *in vivo* (Kirchsteiger *et al.*, 2009; Muthuramalingam *et al.*, 2009; Pulido *et al.*, 2010).

As mentioned above, 2-Cys Prxs have emerged as key players in the connection of cell redox homeostasis and circadian rhythms (Edgar *et al.*, 2012). However, the biochemical mechanism that allows the oscillation of 2-Cys Prx overoxidation is unknown (Stangherlin and Reddy, 2013). It is assumed that overoxidation is favoured by oscillating oxidizing conditions, while the decrease of the level of overoxidation might be due to Srx activity. However, there are organisms lacking Srx that still show circadian oscillation of 2-Cys Prx overoxidation (Stangherlin and Reddy, 2013). 2-Cys Prxs from chloroplasts undergo overoxidation (Kirchsteiger *et al.*, 2009); in addition, these organelles harbour both NTRC, an efficient system for 2-Cys Prx reduction (Pulido *et al.*, 2010), and Srx (Liu *et al.*, 2006; Rey *et al.*, 2007; Iglesias-Baena *et al.*, 2010), the enzymes that may play a central role determining the redox status of 2-Cys Prxs (Puerto-Galán *et al.*, 2013). Thus, plant chloroplasts constitute an excellent system for elucidating the biochemical mechanisms controlling any oscillation of 2-Cys Prx overoxidation and its relationship with the redox status of this organelle.

In this work we have analysed the contribution of NTRC and Srx to the redox status of chloroplast 2-Cys Prxs by a combination of genetic and biochemical approaches. Moreover, we have analysed whether the *NTRC* and *SRX* genes are involved in determining any oscillation of 2-Cys Prx overoxidation in plant chloroplasts. To that end, we have generated an *ntrc-srx* double mutant of *Arabidopsis*, the phenotype of which, as compared with the *srx* and *ntrc* single mutants, shows that the deficiency of NTRC exerts a dominant effect over the deficiency of Srx on plant performance. This notion was further supported by studies *in vitro* showing that the reduction of the disulfide bridge linking the peroxidatic and resolving cysteines is necessary for the overoxidation of plastidial 2-Cys Prxs. In addition, an NTRC-independent, light-dependent component contributing to the redox status of chloroplast 2-Cys Prxs was uncovered. Overall, our data suggest that 2-Cys Prx overoxidation in plant chloroplasts responds to light rather than to circadian oscillations.

## Materials and methods

### Growth conditions and plant material

*Arabidopsis thaliana* wild-type (ecotype Columbia) and mutant plants were routinely grown in soil in growth chambers under long-day (16-h light/8-h dark) or short-day (8-h light/16-h dark) conditions at 22°C during the light and 20°C during the dark and a light intensity of 140 µE m^–2^ s^–1^. The *ntrc* mutant, SALK_012208, has previously been reported (Serrato *et al.*, 2004). A homozygous line, SALK_05324 (Alonso *et al.*, 2003), with a T-DNA insertion at the single gene encoding Srx (AT1G31170) from *Arabidopsis*, here termed the *srx* mutant, was selected by PCR analysis with the oligonucleotides described in Supplementary Table S1. These *ntrc* and *srx* single mutants were used to obtain the *ntrc-srx* double mutant by manual crossing. Seeds resulting from this cross were checked for heterozygosity of the T-DNA insertions in the *NTRC* and *SRX* genes. Plants were then self-crossed and double homozygous lines were identified in the progeny by PCR analysis of genomic DNA using oligonucleotides described in Supplementary Table S1. Seeds were surface sterilized using chlorine gas for 16h, plated on germination media, Murashige and Skoog medium (Duchefa), pH 5.8 containing 0.6% Gelrite (Duchefa) and 0.5% (w/v) sucrose and stratified at 4°C for 2–3 days. For circadian experiments, seedlings were grown for 10 days under long-day conditions and harvested at 4-h intervals during a period of 24h (16-h light/8-h dark), which was followed by a second 24-h period under continuous light or continuous darkness. Samples were immediatelyfrozen in liquid nitrogen and kept at –80ºC until required.

### RNA extraction and RT-qPCR analysis

Total RNA was extracted using Trizol reagent (Invitrogen). cDNA synthesis was performed with 5 µg of total RNA using the Maxima first-strand cDNA synthesis kit (Fermentas) according to manufacturer’s instructions. Real-time quantitative PCR (RT-qPCR) was performed using an IQ5 real-time PCR detection system (Bio-Rad). A standard thermal profile (95°C, 3min; 40 cycles at 95°C for 10 s, and 60°C for 30 s) was used for all reactions. After the PCR, a melting curve analysis (55–94°C at 0.5°C/30s.) was performed to confirm the specificity of the amplicon and to exclude primer-dimers or non-specific amplification. Oligonucleotides used for RT-qPCR analyses are described in Supplementary Table S2. Expression levels were normalized using four reference genes: *ACTIN* and *18S rRNA* (Woodson *et al.*, 2013), *UBIQUITIN* (Ventriglia *et al.*, 2008), and the *Ser/Thr protein phosphatase 2A subunit A3* (*PP2AA3*) (Czechowski *et al.*, 2005). Oligonucleotides for *SRX* gene analyses were designed in exons 4 and 5 of splice variants AT1G31170.1, AT1G31170.2, and AT1G31170.3; the splice variant AT1G31170.4 was not amplified.

### Protein extraction and western blot analysis

For protein extraction, plant tissues were ground with a mortar and pestle in liquid nitrogen. Extraction buffer [100mM Tris-HCl pH 7.9, 10% (v/v) glycerol, 1mM EDTA, 10mM MgCl_2_, 1mM PMSF, and 1% (v/v) protease inhibitor cocktail for plant cell and tissue extracts (Sigma-Aldrich)] was immediately added, and the sample given a swirl on a vortex, and then centrifuged at 13 000*g* at 4°C for 20min. Total protein content was quantified using the Bradford reagent (Bio-Rad) and proteins were subjected to SDS-PAGE, under reducing or non-reducing conditions, as indicated in the figures legends. Western blots were performed as previously described (Kirchsteiger *et al.*, 2009).The anti-2-Cys Prx antibody was previously raised in our laboratory by immunization of rabbits with the full-length recombinant 2-Cys Prx from rice (Pérez-Ruiz *et al.*, 2006). The anti-SO_2/3_ antibody, which was purchased from Abcam (Cambridge, MA, USA), was raised against synthetic sulfonylated peptide corresponding to the active site of human peroxiredoxins I–IV. For blots probed with the anti-SO_2/3_ antibody, membranes were blocked for 30min with 0.5% (w/v) bovine serum albumin (BSA) in TBS supplemented with 0.05% Tween-20 and incubated in the antibody diluted in the same blocking buffer overnight at 4°C. The abundance of 2-Cys Prxs in *Arabidopsis* chloroplasts allowed the redox status of these proteins to be studied in leaf extracts and, thus, isolation of chloroplasts was not necessary.

### Expression and purification of recombinant 2-Cys Prxs A and B and analysis of overoxidation *in vitro*

Recombinant 2-Cys Prxs A and B from *Arabidopsis* were produced in *Escherichia. coli* XL1-Blue with a His-tag at the N-terminus, as previously described (Kirchsteiger *et al.*, 2009). Recombinant proteins were purified from crude extracts of *E. coli* cultures by chromatography in pre-packed Hi-Trap affinity columns (GE Healthcare). Recombinant proteins, 2-Cys Prxs A and B, at a concentration of 0.1 µg µl^–1^ in 100mM phosphate buffer (pH 7.4), 0.5M NaCl, and 10% (v/v) glycerol, were incubated for 30min with or without 20mM DTT and then for another period of 30min with increasing concentrations of H_2_O_2_ (0.1–10mM). Protein samples (0.125 µg of protein) were subjected to SDS-PAGE, under reducing or non-reducing conditions, blotted on nitrocellulose membranes and probed with anti-SO_2/3_ antibody.

### Determination of chlorophylls and the *Fv*/*Fm* ratios

Leaf discs were weighed and frozen in liquid N_2_. After extraction in 1ml methanol for 16h at 4°C, chlorophyll levels were measured spectrophotometrically, as described in Porra *et al.* (1989), and normalized to fresh weight. Room temperature chlorophyll fluorescence was measured using a pulse-amplitude modulation fluorometer (DUAL-PAM-100, Walz, Effeltrich, Germany). The maximum quantum yield of PSII was assayed after incubation of plants in the dark for 30min by calculating the ratio of the variable fluorescence, *Fv*, to maximal fluorescence, *Fm* (*Fv*/*Fm*).

## Results

### NTRC exerts a dominant effect over Srx on 2-Cys Prx overoxidation

**Fig. 1.**
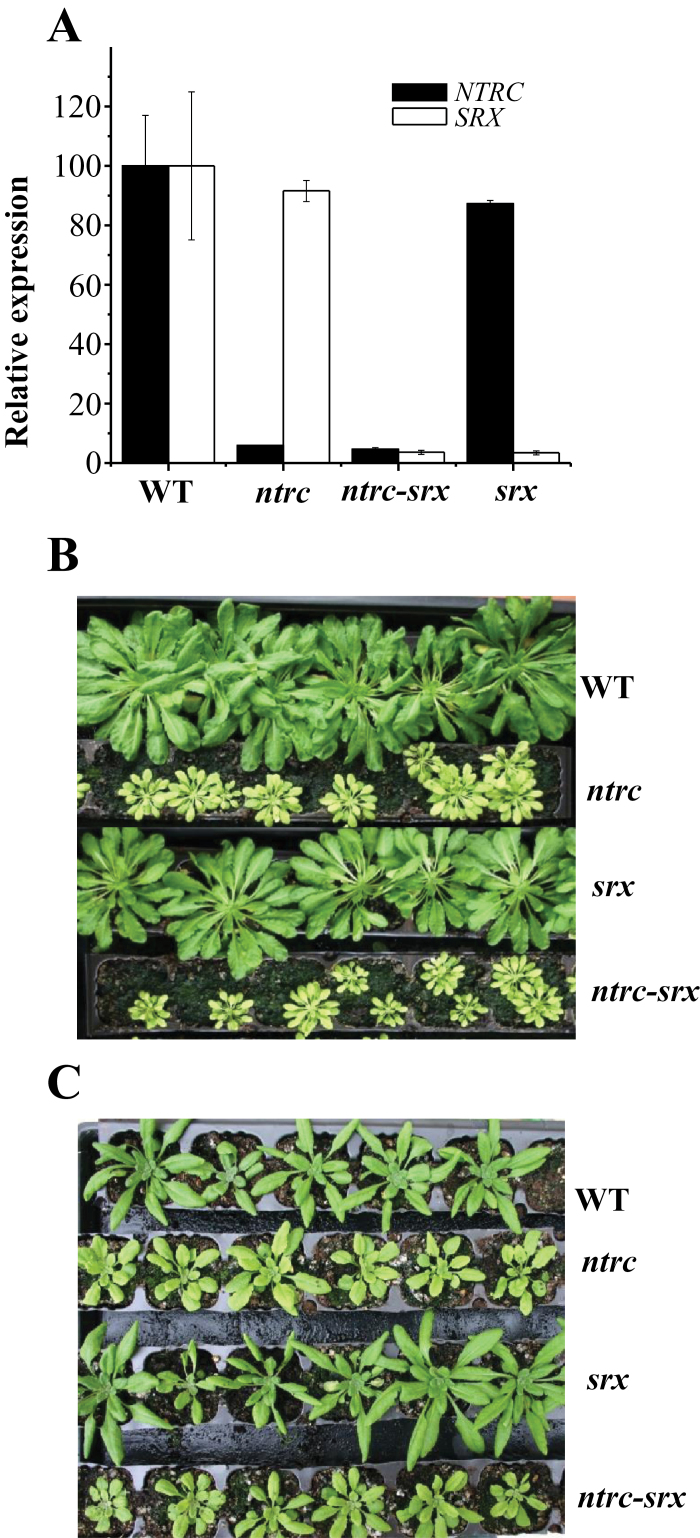
Characterization of the *ntrc-srx* double mutant. The *ntrc-srx* double mutant was obtained by manual crossing of the *ntrc* and *srx* single mutants. (A) The content of *NTRC* and *SRX* transcripts in the *Arabidopsis* lines, as indicated, was determined by RT-qPCR. The amount of *NTRC* and *SRX* transcripts was represented as arbitrary units relative to transcript abundance in wild-type plants, which was set at 100. Analyses were performed three times on two independent biological samples, and the mean values ± SE are indicated. (B) Plants of the different lines grown for 60 days under a short-day photoperiod. (C) Plants of the different lines grown for 32 days under a long-day photoperiod. This figure is available in colour at *JXB* online.

**Fig. 2.**
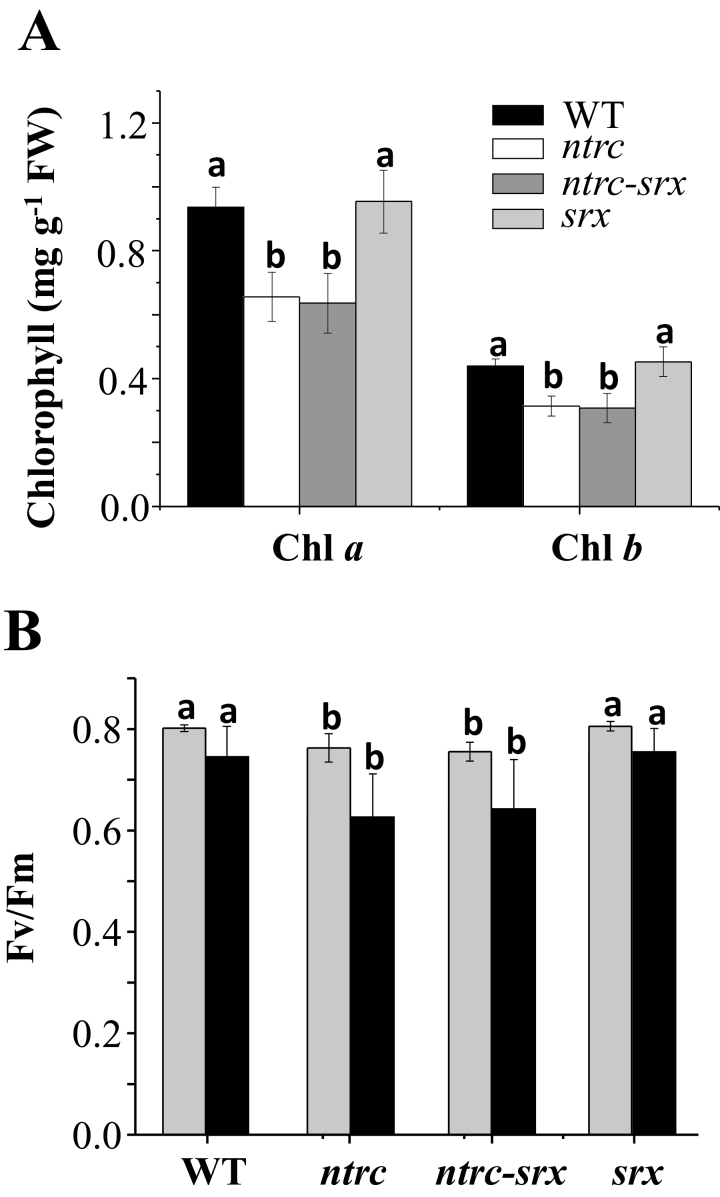
The *ntrc-srx* double mutant and the *ntrc* mutant show similar phenotypes. (A) Chlorophylls *a* and *b* were extracted from rosette leaves of *Arabidopsis* plants that were grown for 30 days under a long-day photoperiod. Determinations were performed on at least three independent biological samples with similar results. Chlorophyll determination was performed twice per sample and mean values ± SE are shown. Letters above bars indicate significant differences (*P <* 0.05) according to Tukey’s multiple range test. (B) Photochemical efficiency of PSII, *Fv*/*Fm*, was determined on 6–8 leaves of the different lines grown under long-day conditions (grey bars) or subjected to a period of 3 days of continuous darkness (black bars). The experiment was performed three times with similar results and mean values ± SE are shown. Letters above bars indicate significant differences (*P <* 0.05) as determined by Tukey’s multiple range test comparing the four *Arabidopsis* genotypes for each condition.

Previous analyses have shown that the level of overoxidation of plastidial 2-Cys Prxs is highly dependent on NTRC (Kirchsteiger *et al.*, 2009) and Srx (Iglesias-Baena *et al.*, 2010), but the contribution of these activities to maintain the redox status of 2-Cys Prxs is not yet known. To address this question an *Arabidopsis* double knockout mutant deficient in NTRC and Srx was obtained by manual crossing of the respective single mutants. The double mutant, termed *ntrc-srx*, was effectively deficient in both transcripts as shown by RT-qPCR analysis (Fig. 1A). In agreement with previous results (Liu *et al.*, 2006; Rey *et al.*, 2007; Iglesias-Baena *et al.*, 2010), the *srx* mutant showed a visual phenotype very similar to wild-type plants, whereas the *ntrc* mutant showed the characteristic growth retarded, pale-green leaf phenotype (Fig. 1B, C), as previously described (Serrato *et al.*, 2004; Pérez-Ruiz *et al.*, 2006; Lepistö *et al.*, 2009). The phenotype of the *ntrc-srx* double mutant is very similar to the *ntrc* mutant and is also sensitive to photoperiod, short-day conditions resulting in a more severe effect (Fig. 1B) than long-day conditions (Fig. 1C). The contents of chlorophylls *a* and *b*, which were characteristically lower in leaves of *ntrc* mutant plants, were decreased at the same level in the *ntrc-srx* double mutant, in contrast with the *srx* mutant, which showed wild-type levels of these pigments (Fig. 2A). Finally, a treatment of continuous darkness showed a similar effect on photosystem II (PSII) efficiency, as determined by the *Fv*/*Fm* ratio, in the *ntrc-srx* double mutant and the *ntrc* mutant, whereas the *srx* mutant behaved like the wild-type plants (Fig. 2B). Therefore, the phenotypic characteristics analysed here revealed the high similarity of the *ntrc-srx* double mutant and the *ntrc* mutant, whereas the *srx* mutant was similar to the wild type. These results show that the deficiency of NTRC exerts a dominant effect on the deficiency of Srx on plant phenotype.

**Fig. 3.**
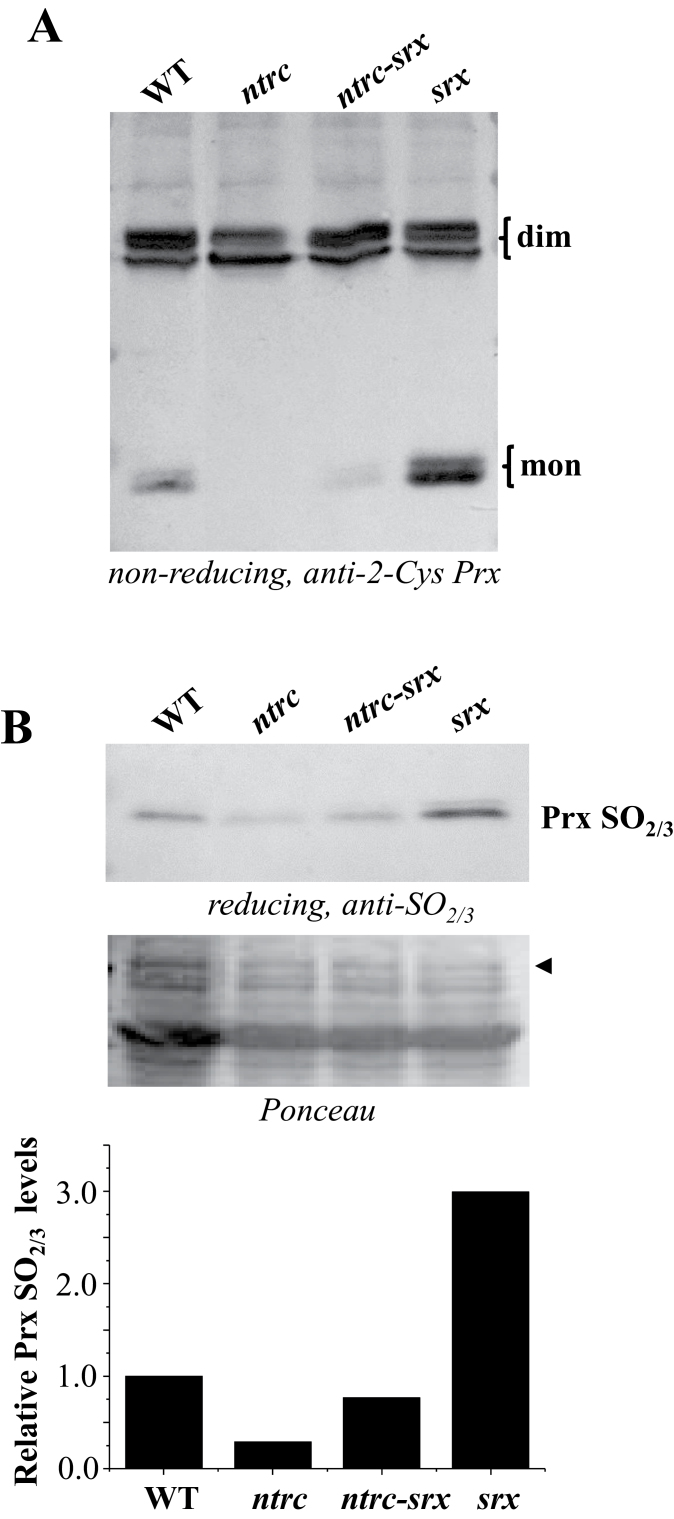
The deficiency of NTRC and Srx has opposite effects on chloroplast 2-Cys Prxs overoxidation. (A) *Arabidopsis* wild-type, *ntrc*, *srx*, and *ntrc-srx* double mutant plants were grown for 10 days under long-day conditions in plates. Protein extracts (12.5 µg) from seedlings were subjected to SDS-PAGE (12% acrylamide), supplemented with 4M urea under non-reducing conditions, electrotransferred onto nitrocellulose filters, and probed with the anti-2-Cys Prx antibody. (B) The amount of overoxidized 2-Cys Prxs was determined on protein extracts (60 µg) from seedlings of the different lines, which were subjected to SDS-PAGE (12% acrylamide), supplemented with 4M urea under reducing conditions. Gels were electrotransferred onto nitrocellulose filters, and probed with the anti-SO_2/3_ antibody. For each genotype, band intensity was quantified with the Scion Image analysis software (Scion Corporation) and normalized against that of the indicated band (arrow) in the ponceau S-stained membrane, which is shown as a loading control. Relative densities are referred to the intensity of the wild type, which was set at 1.

**Fig. 4.**
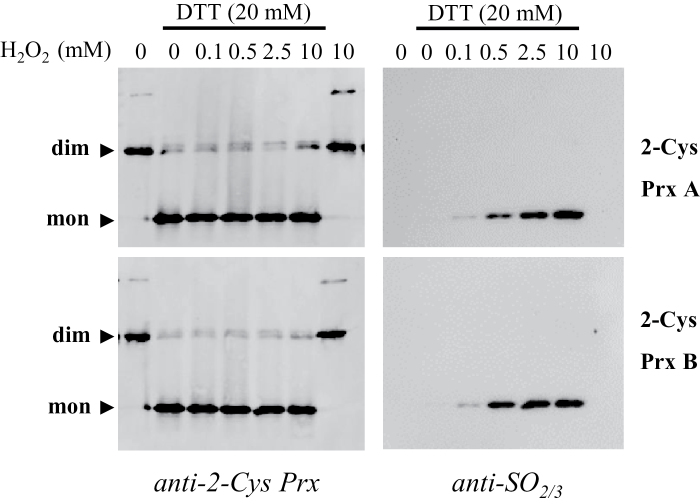
2-Cys Prxs A and B from *Arabidopsis* show similar sensitivity to overoxidation. Purified recombinant 2-Cys Prxs A and B (0.125 μg of protein) from *Arabidopsis* were incubated for 30min with or without 20mM DTT, then samples were incubated in the presence of the indicated concentrations of hydrogen peroxide for 30min. Samples were then subjected to SDS-PAGE (15% acrylamide) under non-reducing conditions, electrotransferred on to nitrocellulose filters and probed with anti-2-Cys Prx or anti-SO_2/3_ antibodies, as indicated. dim, dimer; mon, monomer.

Analysis of the redox status of the chloroplast 2-Cys Prxs, based on non-reducing SDS-PAGE, further supported the hypothesis that the *ntrc-srx* double mutant phenocopies the *ntrc* single mutant. The deficiency of Srx caused the expected increase in the level of monomeric 2-Cys Prxs, reflecting an increase of the overoxidized form of these enzymes (Fig. 3A); this was confirmed by western blot analysis of samples subjected to SDS-PAGE under reducing conditions, with the anti-SO_2/3_ antibody used as a probe (Fig. 3B), in agreement with previous results (Iglesias-Baena *et al.*, 2010). As previously reported (Kirchsteiger *et al.*, 2009), the level of overoxidized 2-Cys Prxs was much decreased in the *ntrc* mutant, as shown by the lower content of the monomeric form of the enzyme, which was almost undetectable (Fig. 3A), and the lower intensity of the band detected with the anti-SO_2/3_ antibody (Fig. 3B). The *ntrc-srx* double mutant also showed reduced levels of overoxidized 2-Cys Prxs, which were slightly, but consistently, higher than in the *ntrc* mutant as shown by both the amount of monomer and the anti-SO_2/3_ antibody (Fig. 3A, B). These results suggest that the key step determining the level of 2-Cys Prx overoxidation is the reduction of the disulfide bridge linking the peroxidatic and resolving cysteines of the enzyme, which is highly dependent on NTRC in plant chloroplasts. To test this possibility further, purified recombinant 2-Cys Prxs A and B from *Arabidopsis* were subjected to treatment with increasing concentrations of hydrogen peroxide. The highest concentration of hydrogen peroxide tested (10mM) did not cause any overoxidation of oxidized 2-Cys Prxs (Fig. 4). In contrast, when 2-Cys Prxs were pre-reduced with DTT, even low concentrations of hydrogen peroxide caused overoxidation, both 2-Cys Prx A and 2-Cys Prx B showing a similar level of sensitivity (Fig. 4). Therefore, these assays *in vitro* show that the disulfide form of these enzymes is resistant to overoxidation.

### Two components, NTRC-dependent and NTRC-independent, contribute to 2-Cys Prx overoxidation in *Arabidopsis* chloroplasts

**Fig. 5.**
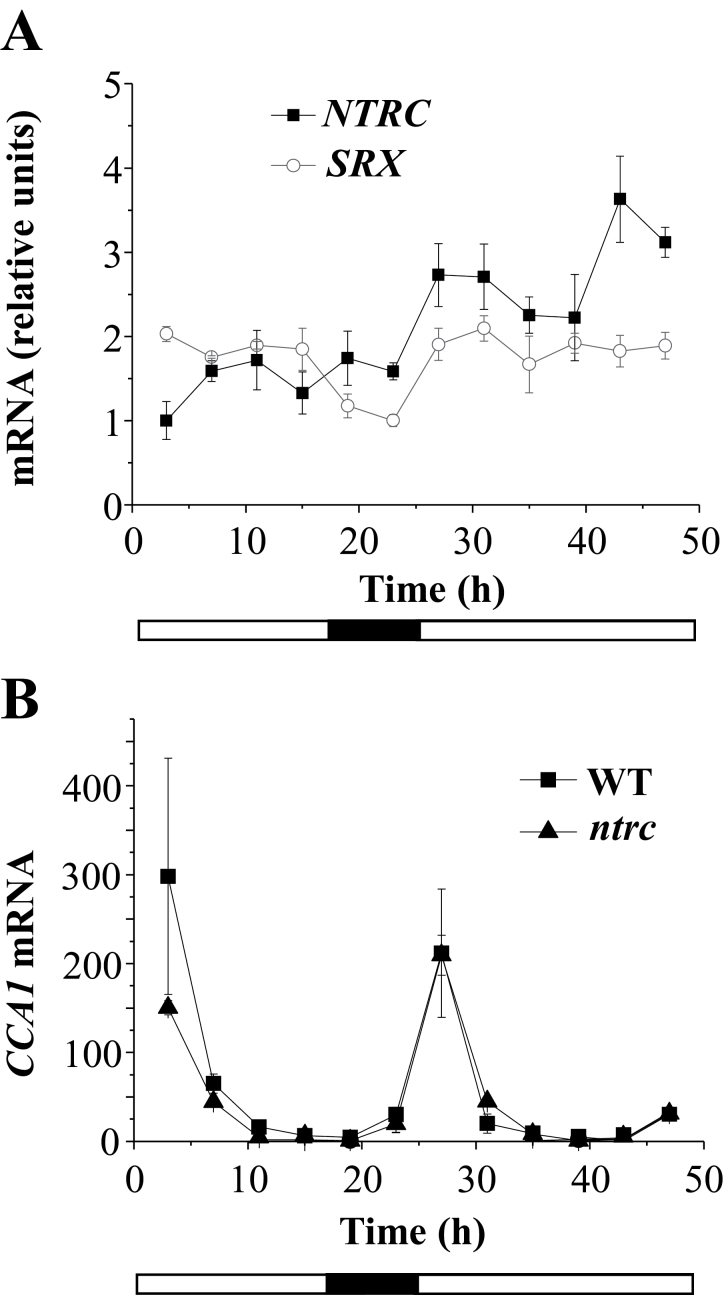
Pattern of expression of the *NTRC* and the *SRX* genes. (A) Wild-type *Arabidopsis* plants were grown under a long-day photoperiod for 10 days, and harvested at 4-h intervals during two consecutive days, the second one with continuous light. The amounts of transcripts of the *NTRC* and *SRX* genes were determined by RT-qPCR and refer to the minimal value for each gene, which was set at 1. (B) *Arabidopsis* wild-type and *ntrc* mutant plants were grown as indicated above and the amount of transcripts of the *CCA1* gene was determined by RT-qPCR and refers to the minimal value, which was set at 1. The experiment was repeated three times with similar results; transcript determinations were performed three times per sample, and the mean values ± SE are indicated.

It has been shown that 2-Cys Prx overoxidation in different organisms undergoes circadian rhythmicity (Edgar *et al.*, 2012), though the biochemical basis of this oscillation is not yet known (Stangherlin and Reddy, 2013). Once the effect of NTRC and Srx on the level of chloroplast 2-Cys Prxs overoxidation was established, we tested whether these enzymes are involved in determining any oscillation of the redox status of 2-Cys Prxs in these plant organelles. To address this question we first analysed the pattern of expression of the *NTRC* and *SRX* genes in *Arabidopsis* during a 24-h period under a long-day photoperiod (16-h light/8-h dark) followed by a period of 24h in continuous light. To that end, seedlings were harvested at 4-h time intervals and the content of *NTRC* and *SRX* transcripts was determined by RT-qPCR. The accumulation of *NTRC* transcripts did not show any significant variation during the first 24-h period and, though moderately increased during the period of continuous light, no circadian oscillation was observed (Fig. 5A). The pattern of expression of the *SRX* gene was very similar to the *NTRC* gene as the amount of *SRX* transcripts showed a slight increase during the light periods but no circadian oscillation was observed (Fig. 5A). The expression profile of both *NTRC* and *SRX* genes is in clear contrast with the pattern of expression of the *CIRCADIAN CLOCK ASSOCIATED 1* (*CCA1*) gene, which was included in these analyses as a positive control of circadian clock-regulated gene expression (Fig. 5B). Moreover, the circadian expression of the *CCA1* gene was not impaired in the *ntrc* mutant, indicating that the deficiency of NTRC does not exert any significant effect on circadian gene expression.

**Fig. 6.**
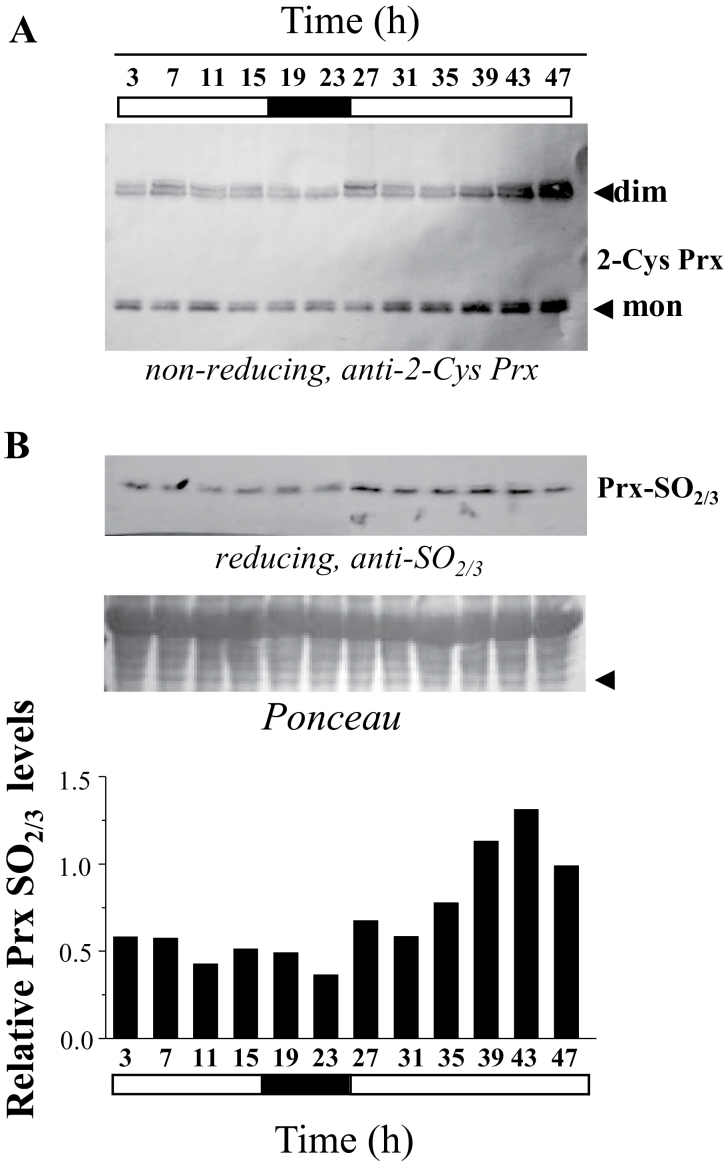
Diurnal pattern of overoxidation of chloroplast 2-Cys Prxs from *Arabidopsis. Arabidopsis* wild-type seedlings that were grown under standard long-day conditions for 10 days were incubated during two consecutive days, the second one with continuous light, and samples were harvested at 4-h intervals, as indicated. (A) Protein extracts (12.5 µg of protein) were subjected to SDS-PAGE (12% acrylamide) supplemented with 4M urea under non-reducing conditions, electrotransferred onto nitrocellulose filters, and probed with the anti-2-Cys Prx antibody. (B) To determine the amount of overoxidized 2-Cys Prxs 60 µg of protein extracts were subjected to SDS-PAGE (12% acrylamide), supplemented with 4M urea under reducing conditions, electrotransferred onto nitrocellulose filters and probed with the anti-SO_2/3_ antibody. For each time point, band intensity was quantified with the Scion Image analysis software (Scion Corporation) and normalized against that of the indicated band (arrow) in the ponceau S-stained membrane, which is shown as a loading control. Relative densities refer to the intensity of the wild type, which was set at 1. dim, dimer; mon, monomer.

**Fig. 7.**
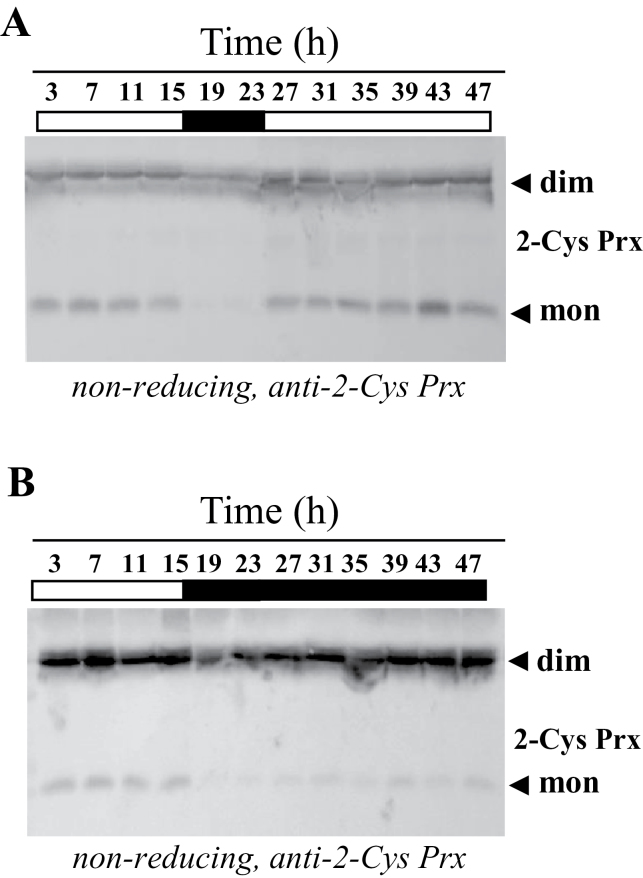
Effect of light on the redox status of chloroplast 2-Cys Prxs from *Arabidopsis ntrc* mutant plants. *Arabidopsis ntrc* mutant seedlings that were grown under standard long-day conditions for 10 days were incubated during two consecutive days, the second one with continuous light (A) or continuous darkness (B) and samples were harvested at 4-h intervals, as indicated. Protein extracts (40 µg of protein) were subjected to SDS-PAGE (12% acrylamide) supplemented with 4M urea under non-reducing conditions, electrotransferred onto nitrocellulose filters, and probed with the anti-2-Cys Prx antibody. dim, dimer; mon, monomer.

Once the pattern of expression of the *NTRC* and *SRX* genes in *Arabidopsis* was established, we analysed the day/night cycling and circadian profiling of chloroplast 2-Cys Prx overoxidation. In order to identify patterns of 2-Cys Prx overoxidation, we studied the amount of monomeric form under non-reducing conditions as well as the accumulation of overoxidized enzyme, revealed by the anti-SO_2/3_ antibody. In wild-type plants, the level of 2-Cys Prx overoxidation did not show any significant oscillation during the first 24-h period as shown by the amount of monomeric enzymes (Fig. 6A) or with the anti-SO_2/3_ antibody (Fig. 6B). During the period of continuous light, the amount of monomer increased indicating the accumulation of overoxidized 2-Cys Prxs (Fig. 6A), which was confirmed with the anti-SO_2/3_ antibody (Fig. 6B). These results suggest that the level of 2-Cys Prx overoxidation in chloroplasts responds to light, rather than to circadian oscillation. To further study the effect of light, the redox status of the 2-Cys Prxs was analysed in the NTRC knockout mutant. As mentioned above (Fig. 2), the level of 2-Cys Prx overoxidation in the *ntrc* mutant was much lower than in wild-type plants. However, when higher amounts of extracts of the *ntrc* mutant were subjected to SDS-PAGE under non-reducing conditions, the monomeric form of the enzymes could be detected allowing analysis of the effect of light on the level of overoxidation of these enzymes (Fig. 7A, B). In the *ntrc* mutant, the monomeric form of the 2-Cys Prxs was detected exclusively during the light period, but not during darkness, thus showing a light-dependent contribution to chloroplast 2-Cys Prx overoxidation (Fig. 7A). Furthermore, the level of monomer was increased during the 24-h period under continuous light (Fig. 7A), but remained low when the first 24-h period was followed by a period of continuous darkness (Fig. 7B). Due to the low level of 2-Cys Prx overoxidation in the *ntrc* mutant, the intensity of the bands detected with the anti-SO_2/3_ antibody was not sensitive enough for an accurate determination and, thus, the level of 2-Cys Prx overoxidation was exclusively based on the amount of monomeric enzymes.

## Discussion

The inactivation of 2-Cys Prxs by overoxidation has been proposed to be a gain of function change which allows important signalling activity of hydrogen peroxide in eukaryotic organisms (Wood *et al.*, 2003). Moreover, transcription-independent rhythmic oscillation of 2-Cys Prx overoxidation in human red blood cells (O’Neill and Reddy, 2011) and the unicellular alga *Ostreococcus tauri* (O’Neill *et al.*, 2011) has led to the proposal that the overoxidation of 2-Cys Prxs is a marker of circadian rhythms (Edgar *et al.*, 2012). Overoxidation is a post-translational modification, which occurs under oxidizing conditions, the reversion of which requires the participation of Srx. Therefore, Srx may exert an important function in the rhythmic oscillation of 2-Cys Prx overoxidation. Interestingly, there are organisms that display oscillation of 2-Cys Prx overoxidation but lack Srx (Stangherlin and Reddy, 2013) and, thus, the actual biochemical mechanism that allows this oscillation remains unknown.

**Fig. 8.**
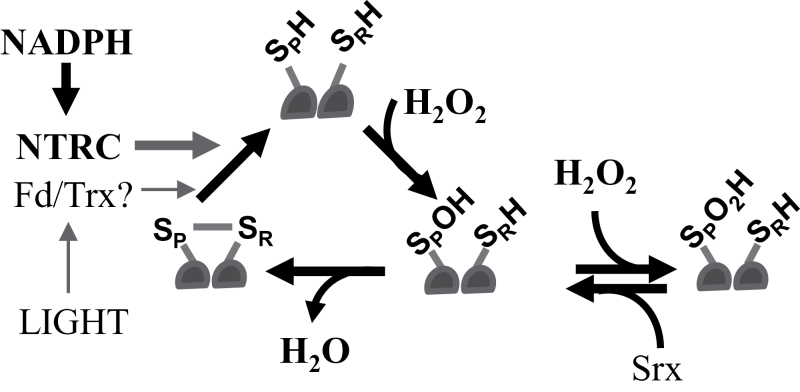
The control of the redox status of chloroplast 2-Cys Prxs. Typical chloroplast 2-Cys Prxs are homodimeric thiol peroxidases containing a peroxidatic (S_P_H) and resolving (S_R_H) cysteine residue in each monomer. The thiolic form of the peroxidatic cysteine attacks the peroxide and becomes transiently oxidized as sulfenic acid (-S_P_OH). This intermediate is then attacked by the resolving cysteine of the other subunit releasing a molecule of water and rendering both cysteines linked by a disulfide bridge. Alternatively, the sulfenic intermediate may be overoxidized to sulfinic acid (-S_P_O_2_H), which is retroreduced by Srx. Previous work has shown that NTRC, which uses NADPH as source of reducing power, is the most relevant reductant of the disulfide-bonded form of 2-Cys Prxs in *Arabidopsis* chloroplasts. In agreement with the latter, the *ntrc-srx* double mutant shows a phenotype similar to the *ntrc* mutant, indicating the relevance of NTRC for determining the level of 2-Cys Prxs overoxidation. Moreover, our results uncovered an NTRC-independent component contributing to 2-Cys Prx overoxidation. The fact that this component is light dependent suggests the participation of the Fd/Trx system.

In this study we analysed the control of 2-Cys Prx overoxidation taking the *Arabidopsis* chloroplast as a model system. The *Arabidopsis* chloroplast harbours two almost identical 2-Cys Prxs, termed A and B, which undergo overoxidation (Kirchsteiger *et al.*, 2009), thus behaving as eukaryotic-type enzymes. It is well known that NTRC, which is an efficient reductant of 2-Cys Prxs, exerts an important role in determining the redox status of these proteins (Pulido *et al.*, 2010). Moreover, Srx, which is a chloroplast-localized enzyme in plants (Liu *et al.*, 2006; Iglesias-Baena *et al.*, 2011), effectively reduces the sulfinic form of platidial 2-Cys Prxs (Iglesias-Baena *et al.*, 2010), indicating that this enzyme might also affect the redox status of 2-Cys Prxs in these organelles (Fig. 8).

To establish the interaction of NTRC and Srx in the control of the redox status of chloroplast 2-Cys Prxs, we generated an *Arabidopsis* double knockout mutant lacking NTRC and Srx. Comparative analysis of the phenotypes of the *ntrc-srx* double mutant with those of the respective *ntrc* and *srx* single mutants clearly indicated the similarity of the double mutant and the *ntrc* mutant, in contrast with the phenotype of the *srx* mutant, which resembles the wild-type plants (Figs 1 and 2). These results show that the deficiency of NTRC has a dominant effect over the deficiency of Srx on plant phenotype, and point to the reduction of the disulfide bridge linking the peroxidatic and resolving cysteines of the 2-Cys Prxs as a key step determining the level of overoxidation of these enzymes. The dominant effect of NTRC was confirmed by analysis of the redox status of plastidial 2-Cys Prxs in these mutants. The deficiency of Srx provoked the expected higher level of overoxidized 2-Cys Prx (Fig. 3), in agreement with previous results (Iglesias-Baena *et al.*, 2010). Interestingly, impairment of the redox status of the 2-Cys Prx caused by Srx deficiency has almost no phenotypic effect since *srx* mutant plants show no visible phenotypic alterations when grown under standard non-stressful conditions (Fig. 1B, C), or in response to treatments of prolonged darkness (Fig. 2B). The impairment of the redox status of the chloroplast 2-Cys Prxs caused by the deficiency of Srx may be relevant under environmental stresses. However, the function of Srx in the response to stress is not yet clear since it has been reported that the *srx* mutant shows susceptibility to oxidative stress (Liu *et al.*, 2006; Iglesias-Baena *et al.*, 2010), but also a higher level of tolerance to photooxidative stress (Rey *et al.*, 2007).

As previously reported (Kirchsteiger *et al.*, 2009; Pulido *et al.*, 2010), the *ntrc* mutant shows a clear deficit of 2-Cys Prx overoxidation (Fig. 3), confirming the primary effect of NTRC on the redox status of these enzymes. However, the finding that the *ntrc-srx* double mutant shows a slightly higher level of 2-Cys Prx overoxidation compared with the *ntrc* mutant (Fig. 3) suggests that there is an NTRC-independent component contributing to 2-Cys Prx overoxidation. A more in-depth analysis of the pattern of 2-Cys Prxs overoxidation in the *ntrc* mutant showed increased overoxidation during the day or under continuous light, while it decreased during the night and remained low during a further 24-h period of continuous darkness (Fig. 7A, B). These results reveal the existence of at least one NTRC-independent redox component contributing to 2-Cys Prxs overoxidation. Because the activity of this component was detected exclusively under illumination, it is probably due to the chloroplast FTR/Trx system, which uses photosynthetically reduced ferredoxin as a source of reducing power (Schürmann and Buchanan, 2008). In this regard, it should be mentioned that 2-Cys Prxs are reduced *in vitro* by Trx *x* (Collin *et al.*, 2003) and CDSP32 (Broin *et al.*, 2002). Moreover, analyses *in vivo* showed impairment of the redox status of 2-Cys Prxs in potato plants lacking CDSP32 (Broin and Rey, 2003), and rice Trx *m* knockdown plants (Chi *et al.*, 2008). Therefore, Trxs *x* and *m*, as well as the Trx-like protein CDSP32, are candidates to account for the NTRC-independent reduction of 2-Cys Prxs, a prerequisite for the subsequent overoxidation of these enzymes, but determining the contribution of these Trxs still requires further work.

Both the genetic and biochemical analyses reported here indicate that a pre-requisite for the overoxidation of chloroplast 2-Cys Prxs is the reduction of the disulfide bridge linking the peroxidatic and resolving cysteine residues at the active site of these enzymes. This is in line with studies showing that structural motifs limiting the formation of this disulfide bridge, as occurs in enzymes from eukaryotic organisms, provoke sensitivity to overoxidation (Wood *et al.*, 2003). The analysis of 2-Cys Prx 3 from human mitochondria, which is resistant to overoxidation, confirmed that disulfide formation protects it from inactivation (Haynes *et al.*, 2013). Furthermore, the endoplasmic reticulum-localized Prx IV shows a low sensitivity to overoxidation, which is due to the lack of a robust Prx recycling system in this cellular compartment (Cao *et al.*, 2014). Thus, the reduction of the disulfide bridge allows a new catalytic cycle of the enzyme with the formation of the sulfenic intermediate, which may be overoxidized to sulfinic acid (Fig. 8). The severe decrease of the level of 2-Cys Prx overoxidation in the *ntrc* and the *ntrc-srx* mutants (Fig. 3) supports the relevance of the disulfide reduction step and, thus, of NTRC in determining the redox status of these enzymes. Once the 2-Cys Prxs become overoxidized, the reversion step catalysed by Srx may be important for adjusting the level of overoxidized enzymes. However, the low phenotypic impact of the deficiency of Srx indicates that this component of the redox regulation of 2-Cys Prxs is less relevant than the disulfide reduction step, at least under standard non-stressful conditions. In this regard, it should be noted that the fraction of overoxidized 2-Cys Prxs was small even in the *srx* mutant (Fig. 3). Since reversion of overoxidation is so far considered to be exclusively catalysed by Srx, the *de novo* synthesis of plastidial 2-Cys Prxs may exert a relevant function in the maintenance of the level of overoxidation of these enzymes.

Based on the results reported here, we established the hypothesis that NTRC and Srx might exert key roles in any oscillation of chloroplast 2-Cys Prx overoxidation. However, neither the analysis of the redox status of the 2-Cys Prxs by non-reducing gels (Fig. 6A) nor the identification of overoxidized 2-Cys Prxs with the anti-SO_2/3_ antibody (Fig. 6B) showed rhythmic oscillation of chloroplast 2-Cys Prx overoxidation, in contrast with previous results (Edgar *et al.*, 2012). Our data, however, are in line with the notion that 2-Cys Prx overoxidation may reflect the oxidant status of the chloroplast. According to this, the low variation of the level of expression of the *NTRC* gene, which does not show circadian oscillation (Fig. 5A), would allow a basal level of 2-Cys Prx reduction and overoxidation, as detected in wild-type plants (Fig. 6A, B). During the day, light would allow additional reduction of the 2-Cys Prxs and, thus, of the level of overoxidation, most probably through the light-dependent FTR/Trx system. The fact that this component is detected exclusively in the absence of NTRC (Fig. 7A, B) suggests that it has a low contribution to the total level of 2-Cys Prxs overoxidation. The pattern of expression of the *SRX* gene, which is slightly increased under illumination (Fig. 5A), might be a mechanism to equilibrate the excess of overoxidation of 2-Cys Prxs produced during the light period. It should be emphasized that light promotes hydrogen peroxide release from the chloroplast (Mubarakshina *et al.*, 2010), which might be an important component of the signalling activity of this organelle. Moreover, 2-Cys Prxs have been proposed to play a central role determining the signalling activity of the chloroplasts (König *et al.*, 2012). Thus, the mechanisms described here allowing the precise tuning of 2-Cys Prx overoxidation in chloroplasts may be central for the signalling activity of these organelles in response to environmental factors such as light or darkness.

## Conclusion

It is well established that NTRC and Srx exert a great influence on chloroplast 2-Cys Prx overoxidation; the relationship between these activities in the control of the level of 2-Cys Prxs overoxidation, however, is as yet unknown. Here, we analysed this relationship by the generation of a double mutant of *Arabidopsis*, *ntrc-srx*, deficient in both NTRC and Srx. The dominant effect of the deficiency of NTRC over the deficiency of Srx suggests that the reaction catalysed by NTRC, i.e. the reduction of the disulfide-linking peroxidatic and resolving cysteine residues, is a pre-requisite for 2-Cys Prx overoxidation. This conclusion was further supported by the finding that this disulfide protects the peroxidatic cysteine from overoxidation *in vitro*. Analyses of plants grown under a long-day photoperiod followed by a 24-h period of continuous light or continuous darkness revealed no circadian oscillation of 2-Cys Prx overoxidation in *Arabidopsis* chloroplasts. In line with these observations, neither the *NTRC* nor the *SRX* genes show circadian regulation of expression. The analysis of 2-Cys Prx overoxidation in the *ntrc* mutant uncovered an NTRC-independent component contributing to 2-Cys Prx overoxidation in *Arabidopsis* chloroplasts. The fact that this component is light dependent, together with the increase of overoxidazed 2-Cys Prxs in response to continuous light, lends further support to the notion that light, rather than the circadian clock, promotes 2-Cys Prx overoxidation. In addition, these findings suggest that the light-dependent Fd/FTR/Trx redox system may contribute to the overoxidation of chloroplast 2-Cys Prxs. Research to identify the Trx(s) involved is now under way.

## Supplementary material

Supplementary Table S1. Oligonucleotides used for genotyping the *srx* and *ntrc* mutants.

Supplementary Table S2. Oligonucleotides used for qRT-PCR analysis.

## Funding

This work was supported by European Regional Development Fund-cofinanced grants from the Spanish Ministry of Economy and Competitivity (MINECO) (BIO2013-43556-P) and Junta de Andalucía (BIO-182 and CVI-5919). A post-doctoral Juan de la Cierva contract from MINECO to J.M.P.-R., and a pre-doctoral fellowship from Junta de Andalucía (Spain) to M.G. are deeply appreciated.

## Supplementary Material

Supplementary Data
