# Supplementary material for: The contribution of NADPH thioredoxin reductase C (NTRC) and sulfiredoxin to 2-Cys peroxiredoxin overoxidation in Arabidopsis thaliana chloroplasts
Source: J Exp Bot. 2015 Jan 5;66(10):2957–66. doi: 10.1093/jxb/eru512 (PMC4423512; doi:10.1093/jxb/eru512)
Supplement: Supplementary Data [file supp_66_10_2957__index.html]

The contribution of NADPH thioredoxin reductase C (NTRC) and sulfiredoxin to 2-Cys peroxiredoxin overoxidation in Arabidopsis thaliana chloroplasts — The contribution of NADPH thioredoxin reductase C (NTRC) and sulfiredoxin to 2-Cys peroxiredoxin overoxidation in Arabidopsis thaliana chloroplasts — Supplementary Data 

# The contribution of NADPH thioredoxin reductase C (NTRC) and sulfiredoxin to 2-Cys peroxiredoxin overoxidation in *Arabidopsis thaliana* chloroplasts

## Supplementary Data

Data files

**Files in this Data Supplement:**

- Supplementary Data - Supplementary Data
