# Supplementary material for: The contribution of NADPH thioredoxin reductase C (NTRC) and sulfiredoxin to 2-Cys peroxiredoxin overoxidation in Arabidopsis thaliana chloroplasts
Source: J Exp Bot. 2015 Jan 5;66(10):2957–66. doi: 10.1093/jxb/eru512 (PMC4423512; doi:10.1093/jxb/eru512)
Supplement: Supplementary Data [file supp_eru512_jexbot136846_file001.pdf]

Table S1. Oligonucleotides used for genotyping of the *srx* and *ntrc* mutants.

Mutant	Locus	Sequence
	Lba1 (T-DNA)	5'-TGGTTCACGTAGTGGGCCATCG-3'
srx (SALK_015324)	At1g31170	5'-ATTTGGATCCTCTAGCGGCGGTGTAGGAC-3'<br>5'-GAGACTGCAGTTCAGCGAAGATGATGCCT-3'
ntrc (SALK_012208)	At2g41680	5'-TCACCAACATGTGGCCC-3'<br>5'-TTCTTCATCTTCACACCCGA-3'

Table S2. Oligonucleotides used for RT-qPCR analysis.

Gene	Locus	Sequence
PP2AA3	At1g13320	5'-GGTTAGATTATTGGCTGTTGAGG-3'<br>5'-CGAGAAATTGACAATCACAGGA-3'
ACTIN	At3g18780	5'-GCACTTGCACCAAGCAGCAT-3'<br>5'-CCTTTCAGGTGGTGCAACGAC-3'
18S rRNA	At3g41768	5'-TATAGGACTCCGCTGGCACC-3'<br>5'-CCCGGAACCCAAAAACTTTG-3'
UBQ	At4g05320	5'-GAAGTTCAATGTTTCGTTTCATGT-3'<br>5'-GGATTATACAAGGCCCCAAAA-3'
CCA1	At2g46830	5'-AACTTCACAGCTCAGTTAACAC-3'<br>5'-AATCCTGTGTTTCTCTTCTCCTC-3'
NTRC	At2g41680	5'-TGAAGATGAAGAAAGAGTACCGAG-3'<br>5'- GGTGTCCTCATTTATTGGCCT-3'
SRX	At1g31170	5'-AGCTAGGGCTTCCAACTATACG-3'<br>5'- CTCTTCAGCGAAGATGATGCC-3'
